# Supplementary material for: Differing Nutrient Intake and Dietary Patterns According to the Presence of Hyper-Low-Density Lipoprotein Cholesterolemia or Hypertriglyceridemia
Source: Nutrients. 2021 Aug 28;13(9):3008. doi: 10.3390/nu13093008 (PMC8469560; doi:10.3390/nu13093008)
Supplement: Supplementary file 1 [file nutrients-13-03008-s001.zip › nutrients-1352636-supplementary.pdf]

Supplementary Table S1. Korean Healthy Eating Index components and standards for scoring.

Classification (No. of components)	Components	Score range	Standard for maximum score	Standard for minimum score
Adequacy (8)	Have breakfast*	0-10	5-7 times/w	0 times/w
	Mixed grains intake*,†	0-5	≥ 0.3 serving/d	0 serving/d
	Total fruits intake†	0-5	• Men aged 19-64 years: ≥ 3 serving/d<br>• Men aged 65 years and overs: ≥ 2 serving/d<br>• Women aged 19-64 years: ≥ 2 serving/d<br>• Women aged 65 years and overs: ≥ 1 serving/d	0 serving/d
	Fresh fruits intake*,†	0-5	• Men aged 19-64 years: ≥ 1.5 serving/d<br>• Women aged 19-64 years: ≥ 1 serving/d<br>• Men aged 65 years and overs: ≥ 1 serving/d<br>• Women aged 65 years and over: ≥ 0.5 serving/d	0 serving/d
	Total vegetables intake	0-5	• Men and women aged 19-64 years: ≥ 8 serving/d<br>• Men aged 65 years and overs: ≥ 8 serving/d<br>• Women aged 65 years and overs: ≥ 6 serving/d	0 serving/d
	Vegetables intake excluding Kimchi and pickled vegetables intake*,†		• Men and women aged 19-64 years: ≥ 5 serving/d<br>• Men aged 65 years and overs: ≥ 5 serving/d<br>• Women aged 65 years and overs: ≥ 3 serving/d	0 serving/d
	Meat, fish, eggs and beans intake†	0-5	Men aged 19-64 years: ≥ 5 serving/d<br>• Women aged 19-64 years: ≥ 4 serving/d<br>• Men aged 65 years and overs: ≥ 4 serving/d	0 serving/d

			• Women aged 65 years and overs: ≥ 2.5 serving/d	
	Milk and milk products intake†	0-10	≥ 1 serving/d	0 serving/d
Moderation (6)	Percentage of energy from saturated fatty acid†,‡	0-10	≤ 7% of total energy intake	> 10% of total energy intake
	Sodium intake†,§	0-10	≤ 2,000 mg/d	> 6,500 mg/d
	Percentage of energy from sweets and beverages†	0-10	≤ 10% of total energy intake	> 20% of total energy intake
	Percentage of energy from carbohydrate†,§	0-5	55-65% of total energy intake	< 50% or > 75% of total energy intake
	Percentage of energy intake from fat†,‡,§	0-5	15-30% of total energy intake	< 10% or > 35% of total energy intake
	Energy intake†,§	0-5	75-125% of the estimated energy intake requirement (EER)	< 60% or > 140% of EER

* Dietary guidelines for Korean adults

† Dietary Reference Intake for Koreans 2015

‡ Recommendation criteria of WHO/FAO

§ 15 or 85 percentile value in Korean adults aged 19 years and over

§ sodium intake, the proportion of participants who intake sodium exceeding 2000mg/day, Korean Nutrition Standard, is so high, therefore, most of participants received 0 points when HEI was evaluated based on 2,000mg criteria. To supplement the distribution of evaluation scores and the accuracy of evaluation indicators, the 85th percentile value of the sodium intake distribution of the participants was set as the minimum reference point by reflecting the current actual intake of Koreans.
